# The Metabolic Role of Gut Microbiota in the Development of Nonalcoholic Fatty Liver Disease and Cardiovascular Disease

**DOI:** 10.3390/ijms17081225

**Published:** 2016-07-29

**Authors:** Marco Sanduzzi Zamparelli, Debora Compare, Pietro Coccoli, Alba Rocco, Olga Maria Nardone, Giuseppe Marrone, Antonio Gasbarrini, Antonio Grieco, Gerardo Nardone, Luca Miele

**Affiliations:** 1 Department of Clinical Medicine and Surgery, Gastroenterology Unit, Federico II University of Naples, 80131 Napoli, Italy; marcosanduzzizamparelli@yahoo.it (M.S.Z.); comparedebora@libero.it (D.C.); pietro.coccoli@unina.it (P.C.); a.rocco@unina.it (A.R.); olga.nardone@libero.it (O.M.N.); 2 Internal Medicine and Gastroenterology Area, Fondazione Policlinico Universitario A. Gemelli, Catholic University of Rome, 00168 Rome, Italy; giusmarrone@gmail.com (G.M.); antonio.gasbarrini@unicatt.it (A.G.); antonio.grieco@unicatt.it (A.G.)

**Keywords:** gut microbiota, cardiovascular disease, NAFLD

## Abstract

The prevalence of metabolic disorders, such as type 2 diabetes (T2D), obesity, and non-alcoholic fatty liver disease (NAFLD), which are common risk factors for cardiovascular disease (CVD), has dramatically increased worldwide over the last decades. Although dietary habit is the main etiologic factor, there is an imperfect correlation between dietary habits and the development of metabolic disease. Recently, research has focused on the role of the microbiome in the development of these disorders. Indeed, gut microbiota is implicated in many metabolic functions and an altered gut microbiota is reported in metabolic disorders. Here we provide evidence linking gut microbiota and metabolic diseases, focusing on the pathogenetic mechanisms underlying this association.

## 1. Introduction

According to the World Health Organization cardiovascular disease (CVD) remains the leading cause of death in Western countries, accounting for about 20 million deaths/year worldwide [1]. Nevertheless, the prevalence of metabolic diseases, such as type 2 diabetes (T2D), obesity, and non-alcoholic fatty liver disease (NAFLD), which are common risk factors for CVD, has dramatically increased over the last decades worldwide.

Both genetic susceptibility and environmental factors contribute to cardio-metabolic pathogenesis. Unfortunately, despite the introduction of new extensive investigation on genetic determinants, such as large-scale genome-wide association study (GWAS), only a few cases can be assigned to genetic factors [2,3]. Thus, the environmental component seems to be primarily involved in CVD burden. 

The largest environmental exposure is food that we take into our intestine in kilogram quantities every day. However, there is an imperfect correlation between dietary habits and the development of metabolic disease. Diet, on the other hand, is known to deeply alter the microbiota composition in the gut. Therefore, recent evidence focused on the role of the gut microbiota in cardio-metabolic disorders and growing interest is aimed to modulate the gut microbiota as a therapeutic strategy.

## 2. Gut Microbiota

The human gut harbors an enormously complex, dynamic, and vast microbial community that is composed mainly of bacteria, but also includes viruses, fungi, protozoa, and archaea [4]. The gut microbiota is estimated to consist of at least 1014 bacteria and archaea, including more than 1100 species and 150-fold more genes than our own host genomes [5].

The introduction of culture-independent methods to study the microbial community revealed the complexity of the gut microbiota. The human gut microbiota is dominated by bacteria belonging to four major phyla: Firmicutes, Bacteroidetes, Actinobacteria (representing >95% of the total microbiota), and Proteobacteria [6]. Interestingly, Actinobacteria and Proteobacteria are more abundant in childhood, while Firmicutes and Bacteroidetes are prevalent in adulthood.

The fetal human gut was supposed to be sterile at birth; however, very recently, it has been reported that there is a passage of microbes between the mother and the fetus through the placenta. The most important colonization of the gastrointestinal tract starts suddenly after the birth and depends on delivery type, maternal flora, environment hygiene, and infant diet. During the first years of life, the gut microbiota composition is widely shifting and changeable, while it stabilizes when the infant reaches 1–3 years of age [7]. Microbial density increases from the proximal to the distal gut, and along the mucosal-lumen axis [8]. Similarly to bacterial density, microbial diversity also increases along the same axis. 

The composition of the gut microbiota is dynamically influenced by several host factors, including diet, lifestyle, antibiotics, and genetic background. In a C57B/L6J mouse model, the use of antibiotics, in early life, alters the host metabolism and adiposity by a modification of the microbiome [9]. Another study described that the maternal administration of antibiotics during the last six months before the pregnancy or the early infancy is related to reduced bacterial diversity of the feces of the neonate, decreased levels of *Lactobacilli* and *Bifidobacteria*, and increased risk of childhood obesity [10].

Diet seems to play a critical role, being linked to quantitative and qualitative modifications of the microbiota composition. A study comparing the gut microbiota of Italian children with those living in a rural African village showed that Bacteroidetes levels (mainly *Prevotella*) were higher in the stool of African children who consumed high amounts of plant polysaccharides while *Enterobacteriaceae* levels were higher in the stool of Italian children [11].

Recently, exercise has also been demonstrated to modulate gut microbiota composition, showing qualitative and quantitative modifications after running [12] in voluntary athletes.

## 3. Gut Microbiota and Energy Balance

### 3.1. Carbohydrate Metabolism

#### 3.1.1. Animal Studies

The gut microbes can benefit the host by extracting energies from otherwise non-digestible carbohydrates and plant polysaccharides via enzymes not encoded by humans [13]. Indeed, indigestible polysaccharides are fermented by colonic microbiota, leading to the generation of short-chain fatty acids (SCFAs) in the form of acetate (60%), propionate (25%), and butyrate (15%). SCFAs are readily absorbed in the colon, where butyrate is an important energy source for colonic epithelial cells, while acetate and propionate reach the liver and peripheral organs, where they are used as substrates for gluconeogenesis [14]. 

SCFAs can also regulate gene expression by acting as signaling molecules, by binding to G-protein-coupled receptors (GPCRs), such as GPR41 (free fatty acid receptor, FFAR3) and GPR43 (FFAR2) [15]. A recent study demonstrated that GPR43-deficient mice, even if fed with a low-fat diet, are obese, while mice overexpressing GPR43 in adipose tissue are lean, even if under a high-fat diet (HFD) [16]. SCFAs-dependent activation of GPR43 can modulate insulin signaling in the adipose tissue, thus preventing fat accumulation. 

Furthermore, SCFAs directly modulate intestinal gluconeogenesis. Propionate affected intestinal gluconeogenesis via the gut-brain neuronal circuit, involving GPR41 and butyrate through cyclic adenosine monophosphate (cAMP)-dependent pathway, independently of GPR43 [17].

As far as butyrate is concerned, it is able to regulate the appetite via the central nervous system, by stimulating the liberation of peptide YY (PYY) and the satietogenic hormones glucagon-like peptide 1 (GLP-1) from enteroendocrine L-cells in the distal small intestine and the colon [18]. PYY is an intestinal hormone [19] known for its ability to decrease intestinal transit rate and increase the harvest of energy from the diet, while GLP-1 improves adipocyte insulin sensitivity and remarkably reduces fat storage in adipose tissue [20]. 

**Figure 1:**
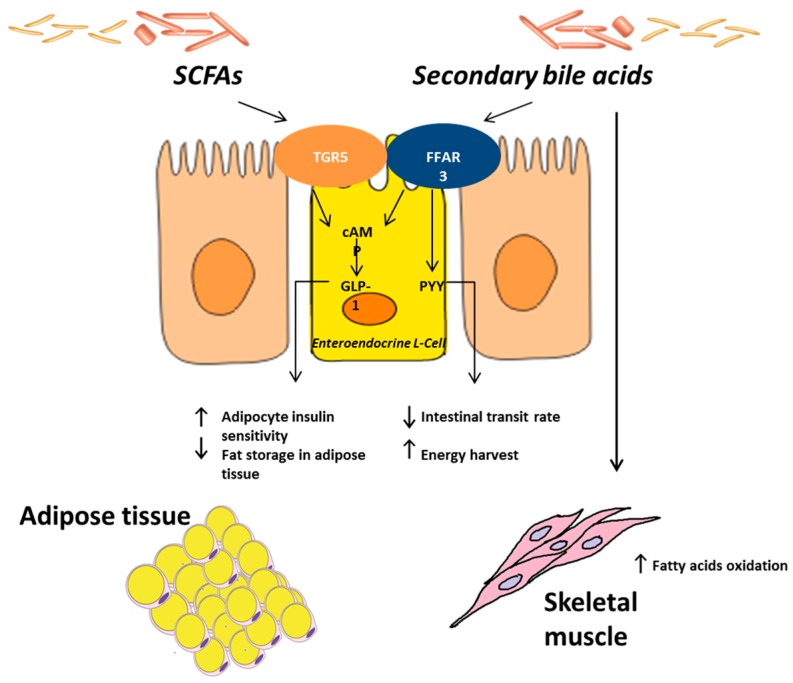
Gut microbiota and energy balance. The gut microbes can benefit the host by extracting energies from otherwise non-digestible carbohydrates and plant polysaccharides via enzymes not encoded by humans. Short-chain fatty acids (SCFAs) modulate intestinal gluconeogenesis via the gut-brain neuronal circuit, involving GPR41 (free fatty acid receptor, FFAR3) and through the cyclic adenosine monophosphate (cAMP)-dependent pathway. Butyrate is able to regulate the appetite in the central nervous system by stimulating the liberation of peptide YY (PYY) and the satietogenic hormone glucagon-like peptide 1 (GLP-1) from enteroendocrine L-cells. PYY decreases the intestinal transit rate and increases the harvest of energy from the diet, while GLP-1 improves adipocyte insulin sensitivity and remarkably reduces fat storage in adipose tissue. Gut microbes can also control the metabolic activity of the host by affecting the composition and the abundance of certain bile acid species. In the ileum microbes deconjugate cholic and chenodeoxycholic acids, which escape intestinal uptake, and are converted into secondary bile acids. Bile acids can also act as signaling molecules by binding cellular receptors such as the bile-acid-synthesis controlling nuclear receptor farnesoid X receptor (FXR), G-protein-coupled receptors (GPCR), and TGR5. While primary bile acids can impair glucose metabolism by binding FXR, secondary bile acids, by binding TGR5, improve glucose homeostasis and increase energy expenditure in skeletal muscle.

Gut microbes can also control the metabolic activity of the host by affecting the composition and the abundance of certain bile acid species [21]. The cholic and chenodeoxycholic acids are produced in the liver from cholesterol, and are needed for the absorption of cholesterol, dietary fats, and fat-soluble vitamins. In the ileum microbes deconjugate these bile acids, which escape intestinal uptake, and are converted into secondary bile acids. Bile acids can also act as signaling molecules by binding cellular receptors, such as the bile-acid-synthesis controlling nuclear receptor farnesoid X receptor (FXR), GPCR, and the G protein-coupled bile acid receptor TGR5 [22]. While primary bile acids can impair glucose metabolism by binding FXR [23], secondary bile acids (deoxycholic and chenodeoxycholic acid) improve glucose homeostasis by binding TGR5 [24] (Figure 1).

Furthermore, animal studies demonstrated that *Akkermansia muciniphila* concentrations are inversely correlated with the body weight and glucose tolerance [25]. Hansen et al. recently demonstrated that vancomycin administration in non-obese diabetic rats increased *A. muciniphila* levels and ameliorated glucose homeostasis [26].

#### 3.1.2. Human Studies

In humans, a metagenome-wide association study demonstrated significant modifications of specific gut microbes and metabolic pathways in T2D patients [27]. The study was performed using stool samples of 344 Chinese patients and showed a diminution of butyrate-producing bacteria, such as *Roseburia intestinalis* and *Faecalibacterium prausnitzii*, and an abundance of *Akkermansia muciniphila*. Recently, an interesting study conducted in Europe on postmenopausal patients with normal, impaired, or diabetic glucose regulation, showed somewhat contradictory results in comparison to the Chinese study. Different techniques, but also ethnic and dietetic factors, can account for the discrepancy [28]. 

A previous, smaller study demonstrated higher levels of *Lactobacillus* spp. in T2D patients in comparison to healthy controls [29] and both Chinese and European studies showed enhanced concentration of *Lactobacillus gasseri*, *Streptococcus mutans* and certain *Clostridiales*, and lower levels of *Roseburia intestinalis* and *Faecalibacterium prausnittzii*, in the diabetic cohort. 

Moreover, certain antidiabetic medications can produce modification of the gut microbiota composition. A recent paper demonstrated that metformin administration resulted in increased levels of *A. muciniphila*, improvement of glucose tolerance, and reduced systemic inflammation [30,31]. In this light, the administration of prebiotics, such as oligofructose, increased *A. muciniphila* concentrations with various metabolically-beneficial outcomes [30]. In this way *A. muciniphila* reveals to be a promising candidate in the understanding of the complex role of intestinal microbes in metabolic disorders even if data are still somewhat controversial.

In summary, evidence suggests that gut microbiota plays a critical role in the energy balance by fermentation of carbohydrates in SCFA. Gut microbiota composition appears to be involved in the pathogenesis of diabetes, but further interventional studies are needed to use it as a diagnostic and therapeutic tool.

### 3.2. Lipid Metabolism

#### 3.2.1. Animal Studies

It is supposed that HFD may affect epithelial integrity, leading to impaired intestinal permeability and, consequently, to metabolic endotoxemia and systemic inflammation [32]. Chronic exposition to low-dose lipopolysaccharide (LPS) in mice led to hepatic fat deposition, insulin resistance (IR), hyperlipidemia, adipose tissue macrophages infiltration, and obesity, similar as after feeding a HFD [33,34]. Furthermore, these effects were abolished when TLR4 (Toll-like receptor, directly involved in LPS binding) knockout mice were fed a HFD, or *ob*/*ob* mice and HFD-fed mice were treated with antibiotics or prebiotics [32]. 

Another complex diet-independent effect of microbiota on host metabolism is mediated by bacterial compounds, such as peptidoglycan and flagellin, that are able to activate inflammatory pathways [35]. For example, TLR5 is a pattern recognizer for flagellin, which can modulate intestinal homeostasis by activating various intracellular signaling pathways. *TLR5−/−* mice show a metabolic phenotype, exhibiting gut microbiome modifications, dyslipidemia, hypertension, IR, and obesity. Similarly to other studies, colonization of germ-free mice with *TLR5−/−* mice caecum microbes, led to a metabolic phenotype [36].

Studies in rodents genetically predisposed to obesity (*ob*/*ob*), first revealed an increase in the Firmicutes/Bacteroidetes ratio [37] and analogous differences were observed in the gut microbiota in human obesity [37,38]. 

Interestingly, it has been clearly shown that the obese phenotype is a transmissible trait, by transplanting the caecal microbiome from obese mice into germ-free mice who developed the same phenotype of the donor [39,40]. Similarly, when germ-free mice were colonized with a caecum-derived microbiome of conventional mice, the amount of the total body fat increased and insulin sensitivity decreased [41]. 

A recent paper showed that colonization of germ-free mice with fecal microbiota from twin pairs discordant for obesity resulted in an obese phenotype in mice receiving microbiota from the obese twin, while mice receiving microbiota from the lean twin showed less weight gain and adiposity [42]. In the same study, authors also showed that cohousing of *ob*/*ob* mice and lean mice led to the acquisition of the gut microbiota of the lean rodents by *ob*/*ob* mice but not to the acquisition by lean mice of the *ob*/*ob* mice microbiota. In addition, cohousing of *ob*/*ob* mice and lean mice fed with high vegetables and fruit, a low-fat diet (LFD), resulted in a greater acquisition of microbes of the lean mice by *ob*/*ob* rodents. 

A recent study showed that the increased levels of acetate in dysbiosis stimulated the parasympathetic nervous system which, in turn, enhanced the secretion of glucose-stimulated insulin and ghrelin, leading to hyperphagia and obesity [43].

Overall, these findings suggest an active role of gut microbiome in the development of obesity, and the major mechanism involved appears to be the more efficient energy harvest from food [44].

#### 3.2.2. Human Studies

Several studies demonstrated that obese patients usually show chronic adipose tissue inflammation and that the obese-associated gut microbiome was related with a high production of pro-inflammatory cytokines [45]. In addition, several, but not all, human adult evidence showed a decrease of the diversity of the microbiota and an increased Firmicutes/Bacteroidetes ratio [46]. Weight loss and a LFD reversed the obese core microbiome versus the healthy microbiota, by increasing the relative proportion of Bacteroidetes spp. and the bacterial diversity [47]. Furthermore, after bariatric surgery, levels of Bacteroidetes and Prevotella negatively correlated with adiposity and energy harvest [48].

Recent data on the gut fungal species of obese humans showed a reduced family biodiversity in obese individuals but no modifications in the richness of the gut mycobiome between obese and healthy subjects [49]. 

In summary, animal and human evidence described an ‘obese core microbiome’ which seems to be involved in the pathogenesis of obesity.

### 3.3. Gut Microbiota and NAFLD

The first evidence for a putative role of the gut microflora in NAFLD was suggested more than 20 years ago in patients with small intestinal bacterial overgrowth (SIBO) who were prone to develop NAFLD, and in a rat model with a blind intestinal loop in which hepatic injury was prevented by antibiotics [50].

An human study showed an increased gut permeability and prevalence of SIBO in NAFLD patients [51]. 

Recent studies focused on the increased endogenous ethanol production by the gut microbiota that is primary involved in NAFLD development [52,53]. Indeed, *ob*/*ob* mice showed an increase in breath ethanol amount, while normal ethanol levels were observed if mice were treated with neomycin [54]. 

Convincing evidence also demonstrated that the microbiota-dependent choline conversion into methylamines in strain 129S6 on a HFD decreases the bioavailability of choline mimicking the effects achieved by choline-deficient diets [55]. In this study, the authors observed that dietary choline depletion induced gut microbiota modification, and that Erysipelotrichi and Gammaproteobacteria levels were associated with changes in liver fat.

A recent study demonstrated that differences in intestinal microbiota composition could explain the varying response to a HFD in mice [56]. In this study, two donor C57BL/6J mice were identified on the basis of their responses to a high-fat diet (HFD); although both groups showed similar body weight gain, one mouse, called the ‘responder’, displayed hyperglycemia and systemic inflammation, whereas the other, called a ‘non-responder’, was normoglycemic. When the microbiota of both groups were transplanted into germ-free mice which were then fed with the same HFD, only mice colonized with ‘responders’ microbiota showed hyperglycemia and fatty liver disease, in absence of systemic and hepatic inflammation.

Moreover, an interventional study on ApoE−/− mice, a genetic model of dyslipidemia, intestinal inflammation, and steatohepatitis, showed that the administration of the probiotic VSL#3 improved insulin resistance, and reduced the aortic plaque extension, mesenteric adipose tissue inflammation, and steatohepatitis [57].

A human study demonstrated that the administration of *Bifidobacterium longum* with fructo-oligosaccharides, plus lifestyle modifications, were able to significantly reduce serum aspartate transaminase (AST) levels, tumor necrosis factor (TNF)-α, CRP (C-reactive protein), HOMA-IR (Homeostasis Assessment Model-Insulin Resistance), serum endotoxin, steatosis, and the non-alcoholic steatohepatitis (NASH) activity index when compared to lifestyle modification alone [58].

Dysbiosis-driven inflammatory response seems to play a major role in the pathogenesis of NAFLD. Intestinal microorganisms have highly conserved molecules, named “pathogen associated molecular patterns” (PAMPs) which are recognized specifically by pattern recognition receptors (PRRs), such as TLRs and nucleotide binding oligomerization domain-like receptors (NLRs). Several studies have shown that PRR stimulation can increase pro-inflammatory cytokines and chemokines by different intracellular signaling cascades, while commensal microbiota are able to counteract this inflammatory response by interfering in TLR-dependent nuclear factor kappa B (NF-κB) transcription [59,60,61]. The multimeric signaling platforms, called ‘inflammasomes’, appear to be primarily involved in the gut microbiota-driven liver steatosis and inflammation. The inflammasome, which is frequently identified by its first sensing molecules, such as NLRP6 and NLRP3 (NOD-like receptors, pyrin domain containing 6 and 3), have common pathways leading to interleukin (IL)-18 and IL-1 activation via caspase-1 activation. Inflammasome-deficient mice showed a reduction of Firmicutes and an increase of Bacteroidetes, associated with increased steatosis and inflammation via TLR4 and TLR9 activation, leading to enhanced hepatic TNF-transcription [62]. Co-housing of inflammasome-deficient mice with wild-type mice resulted in exacerbation of obesity and steatosis, concluding that NLRP6 and NLRP3 inflammasomes negatively regulate NAFLD/NASH progression. Furthermore, co-housing mice defective in TLR4 and TLR with inflammasome-deficient mice did not worsen NAFLD/NASH [63].

In summary, evidences suggest that dysbiosis may play a critical role in the development of NAFLD/NASH by metabolic and inflammatory pathways. 

### 3.4. Cardiovascular Disease

Gut microbiota composition is modified not only in diabetes, obesity, and NAFLD, with adverse cardiovascular outcomes, but also in hypertension. Indeed, animals with hypertension showed decreased gut bacterial richness and diversity with associated reduced levels of acetate and butyrate which negatively correlate with systemic inflammation [64]. Furthermore, several studies showed that the “metabolic endotoxemia” caused by the exposure to LPS promoted a systemic low-grade inflammation with an increased cardiovascular risk [65,66]. Indeed, the binding of TLR4 by LPS, and the activation of an immune response, triggered the release of proinflammatory molecules that promoted endothelial dysfunction, oxidation of low-density lipoproteins (LDLs), thrombogenesis, and the formation and rupture of the atherosclerotic plaque [66].

Population studies supported a relation between infection and CVD, in consideration of higher cardiovascular risk and blood pressure in patients affected by periodontal disease [67,68]. Mounting evidence in animals and in humans is accumulating showing that gut microbiota is associated with CVD [69]. One study failed to show any overall difference in fecal microbiota composition of patients with atherosclerosis, however, gut and atherosclerotic plaque microbiota showed several operational taxonomic units (OTUs) [70]. Indeed, atherosclerotic plaque was shown to harbor its own microbiota dominated by Proteobacteria and Collinsella [71]. 

Furthermore, a meta-analysis of clinical trials of antibiotics therapy in people with atherosclerosis failed to demonstrate their positive effect on cardiovascular mortality [72]. Nowadays, only one prospective randomized trial with azithromycin performed for secondary prophylaxis of coronary disease failed to reduce cardiovascular event rates [73]. 

Metabolomic analyses of plasma samples in humans identified novel metabolites and connecting pathways related with cardiovascular risk [74]. Major differences were found in choline, betaine, and trimethylamine *N*-oxide (TMAO), metabolites linked to phosphatidylcholine (PC) metabolism. Choline is known to be an essential nutrient [75] and its dietary deficiency can lead to muscle damage and liver steatosis. TMAO arises from the bacterial metabolism of choline via an intermediate trimethylamine (TMA), which reaches the liver and is converted into TMAO by oxidizing flavin monooxygenases 3 (FMO3) [76]. TMAO was undetectable in germ-free mice following a PC or carnitine challenge, but conventionalization of the rodents increased TMAO levels [77]. Similarly, when mice were treated with a cocktail of antibiotics, PC administration did not result in TMAO. Oral, but not parental, delivery of phosphatidylcholine was associated to higher levels of TMAO, suggesting that gut metabolic step is required for TMAO production. Interestingly, TMAO administration to apolipoprotein E-null mice was observed to produce macrophage foam cell formation in both the artery wall and the peritoneal cavity and to enhance aortic root atherosclerotic plaque development. 

In a large cohort study of over 4000 patients undergoing elective coronary angiography, TMAO levels were able to predict major adverse cardiac events independently from traditional CV risk factors [78]. Blood choline and carnitine levels were also predictors of major cardiac events with respect to stable coronary artery disease, but only when TMAO levels were concomitantly increased [76]. 

In summary, animal and human evidence suggest that the gut microbiota plays a contributory role in the development of CVD but need further studies to use the gut microbiota as a new target for prevention and treatment of CVD.

## 4. Concluding Remarks

Over the last years, gut microbiota has emerged as a fascinating “new organ”, involved in many intestinal and extra-intestinal pathologies. New sequencing techniques led to the discovery of a huge complexity of the microbiome and to the identification of potential genes involved in gut microbiota-host interaction.

Exciting new data suggest a clear contributory role for gut microbes in cardiometabolic disorders, such as diabetes, obesity, NAFLD/NASH, and atherosclerosis. However, more interest should be paid to the association with metabolomics, metagenomic, and functional studies, supplemented by prospective observational and interventional studies. The exploration of gut microbiota composition and function has now become a field of interest that offers a new frontier to discover the complex human host physiology and disease and providing new biomarkers and innovative therapeutic approaches.
